# Case report: Cryptogenic giant brain abscess caused by *Providencia rettgeri* mimicking stroke and tumor in a patient with impaired immunity

**DOI:** 10.3389/fneur.2022.1007435

**Published:** 2022-09-23

**Authors:** Yu Zhao, Baorong Lian, Xudong Liu, Qizheng Wang, Daxue Zhang, Qi Sheng, Liming Cao

**Affiliations:** ^1^Department of Neurology, Shenzhen Third People's Hospital, Shenzhen, China; ^2^Department of Neurology, The Second Affiliated Hospital, Southern University of Science and Technology, Shenzhen, China; ^3^Shantou University Medical College, Shantou University, Shantou, China; ^4^Department of Neurology, The First Affiliated Hospital of Shenzhen University, Shenzhen, China; ^5^School of Nursing, Anhui Medical University, Hefei, China

**Keywords:** *Providencia rettgeri*, cryptogenic brain abscess, impaired immunity, metagenomic next-generation sequencing, stroke-like onset, brain tumor, case report

## Abstract

The highly lethal cryptogenic brain abscess can be easily misdiagnosed. However, cryptogenic brain abscess caused by *Providencia rettgeri* is rarely reported. We present the case of a cryptogenic *Providencia rettgeri* brain abscess and analyze the clinical manifestations, imaging findings, treatment, and outcome to improve the level of awareness, aid in accurate diagnosis, and highlight effective clinical management. A 39-year-old man was admitted to the hospital after experiencing acute speech and consciousness disorder for 1 day. The patient had a medical history of nephrotic syndrome and membranous nephropathy requiring immunosuppressant therapy. Magnetic resonance imaging revealed giant, space-occupying lesions involving the brain stem, basal ganglia, and temporal-parietal lobes without typical ring enhancement, mimicking a tumor. Initial antibiotic treatment was ineffective. Afterward, pathogen detection in cerebrospinal fluid using metagenomic next-generation sequencing revealed *Providencia rettgeri*. Intravenous maximum-dose ampicillin was administered for 5 weeks, and the patient's symptoms resolved. Cryptogenic *Providencia rettgeri* brain abscess typically occurs in patients with impaired immunity. Our patient exhibited a sudden onset with non-typical neuroimaging findings, requiring differentiation of the lesion from stroke and brain tumor. Metagenomic next-generation sequencing was important in identifying the pathogen. Rapid diagnosis and appropriate use of antibiotics were key to obtaining a favorable outcome.

## Introduction

The brain abscess is a potentially fatal parenchymal brain lesion caused by local infection or remote spread (1). The incidence of brain abscesses is estimated to be 0.3–1.3 per 100,000 persons per year (2). The wide application of antibiotics has contributed to a gradual decline in the prevalence of brain abscesses.

The mortality of brain abscesses decreased from 22.8% in the 1950s to 6.3% in the early 21st century (3). However, cases of cryptogenic brain abscesses with unknown origin and atypical symptoms are increasing (3). The source of infection remains unclear in a significant proportion of brain abscesses, even after thorough investigations, and these are considered cryptogenic brain abscesses (4). Cryptogenic abscesses occur in 4.6–43.4% of cases (4, 5), and they are easily misdiagnosed and have a poor prognosis (1). Early diagnosis and treatment are crucial to minimize complications and reduce mortality (1). *Providencia rettgeri* is a motile, gram-negative, and facultative anaerobic bacterium widely distributed in the environment (6). It can cause zoonotic opportunistic infections, such as urinary tract infections (7), endocarditis (8), and rare intracranial infections (9).

To our knowledge, cryptogenic giant brain abscess caused by *Providencia rettgeri* is rare, and its clinical and imaging features are poorly understood. We present such a case with a literature review to improve the diagnosis and management of this disease.

## Case description

In October 2021, a 39-year-old man was admitted to our hospital after experiencing speech disorder and right hemiplegia for 1 day with consciousness disorder for over 3 h. Emergency non-contrasted brain computed tomography (CT) scan revealed left basal ganglia and temporoparietal finger-shaped edema. The head and neck CT angiography displayed no obvious abnormality. The patient had presented with edema in both lower limbs 3 months previously and based on renal pathology, nephrologists diagnosed membranous nephropathy (stage II) and nephrotic syndrome. Intravenous (IV) cyclophosphamide, oral prednisone (40 mg per day), and oral valsartan were administered. He had no history of infectious or genetic diseases.

Physical examination on admission showed a body temperature of 36.2°C, blood pressure of 173/103 mmHg, confusion, aphasia, shallow nasolabial sulcus on the right side, decreased muscle strength (0/5) in the right limbs, and decreased muscular tension, with no other abnormal neurological or cardiopulmonary findings. The National Institutes of Health Stroke Scale score was 15.

Blood analysis revealed an absence of anti-double-stranded DNA, anti-nucleosome, anti-SM, anti-proliferating cells, *Treponema pallidum*, human immunodeficiency virus, anti-protease 3, anti-myeloperoxidase, and anti-glomerular basement membrane antibodies. Serum creatine kinase, homocysteine, uric acid, ammonia, troponin I, myoglobin, and N-terminal pro-brain natriuretic peptide levels were within normal limits. Glucose and lactic acid levels in cerebrospinal fluid (CSF) on day 2 were normal. Cytotoxic T lymphocyte count and TCD3+ CD4+/ TCD3+ CD8+ were normal. The gene amplification method for detecting cytomegalovirus DNA in plasma gave a negative result. The CSF smear stained with India ink showed no cryptococcus. The test result for cryptococcal capsular antigen in the CSF was negative. Initial CSF culture yielded no pathogens. Metagenomic next-generation sequencing [mNGS, WillingMed Technology (Beijing) Co., Ltd., Beijing City] of the CSF on day 18 revealed 21 sequences of *Providencia rettgeri* (reference range < 8 sequences). A repeat mNGS (the Guangzhou Institute for Respiratory Health, Guangzhou City) showed 14 sequences of *Providencia rettgeri*. Repeat testing of the CSF on day 28 revealed that the white blood cell count and protein level were within normal limits, and the CSF culture yielded no growth. Serum urea and creatinine levels and the glomerular filtration rate on day 34 were normal. The other laboratory results are shown in Table 1.

**Table 1:** Laboratory findings.

**Variable**	**Result**	**Reference range**	**Interpretation**
WBC count, × 10^9^/L	12.05	3.5–9.5	Elevated
Absolute neutrophils, × 10^9^/L	9.78	1.8–6.3	Elevated
Serum total protein, g/L	50.0	63–82	Decreased
Serum albumin, g/L	24.0	35–50	Decreased
Serum urea, mmol/L	10.50	3.1–8.0	Elevated
Serum creatinine, μmol/L	130.3	57–97	Elevated
CRP, mg/L	16.3	0–6	Elevated
Fibrinogen, g/L	5.17	2–4	Elevated
Procalcitonin, ng/mL	0.33	< 0.1	Elevated
Fasting blood glucose, mmol/L	6.9	3.9–6.1	Elevated
Erythrocytes in urine, /μL	1136.6	0–18	Elevated
Protein in urine, g/L	10	Negative	Elevated
Helper T lymphocytes	25.1%	34–52%	Decreased
Plasma Epstein–Barr virus DNA	8.84	5.0*X*10^2^	Elevated
CSF pressure on day 2, mmH_2_O	330	80~180	Elevated
CSF WBC count, × 10*6/L	97	0–8	Elevated
CSF protein, mg/L	605	120–600	Elevated
Fibrinogen on day 3, g/L	6.65	2–4	Elevated
CRP on day 3, mg/L	52.41	0–6	Elevated
Procalcitonin on day 6, ng/mL	0.34	< 0.1	Elevated
D-dimer on day 6, μg/mL	13.83	< 0.5	Elevated
Interterleukin-6 on day 6, pg/mL	25.6	< 0.1	Elevated

CSF, cerebrospinal fluid; CRP, C-reactive protein; WBC, white blood cell.

**Figure 1:**
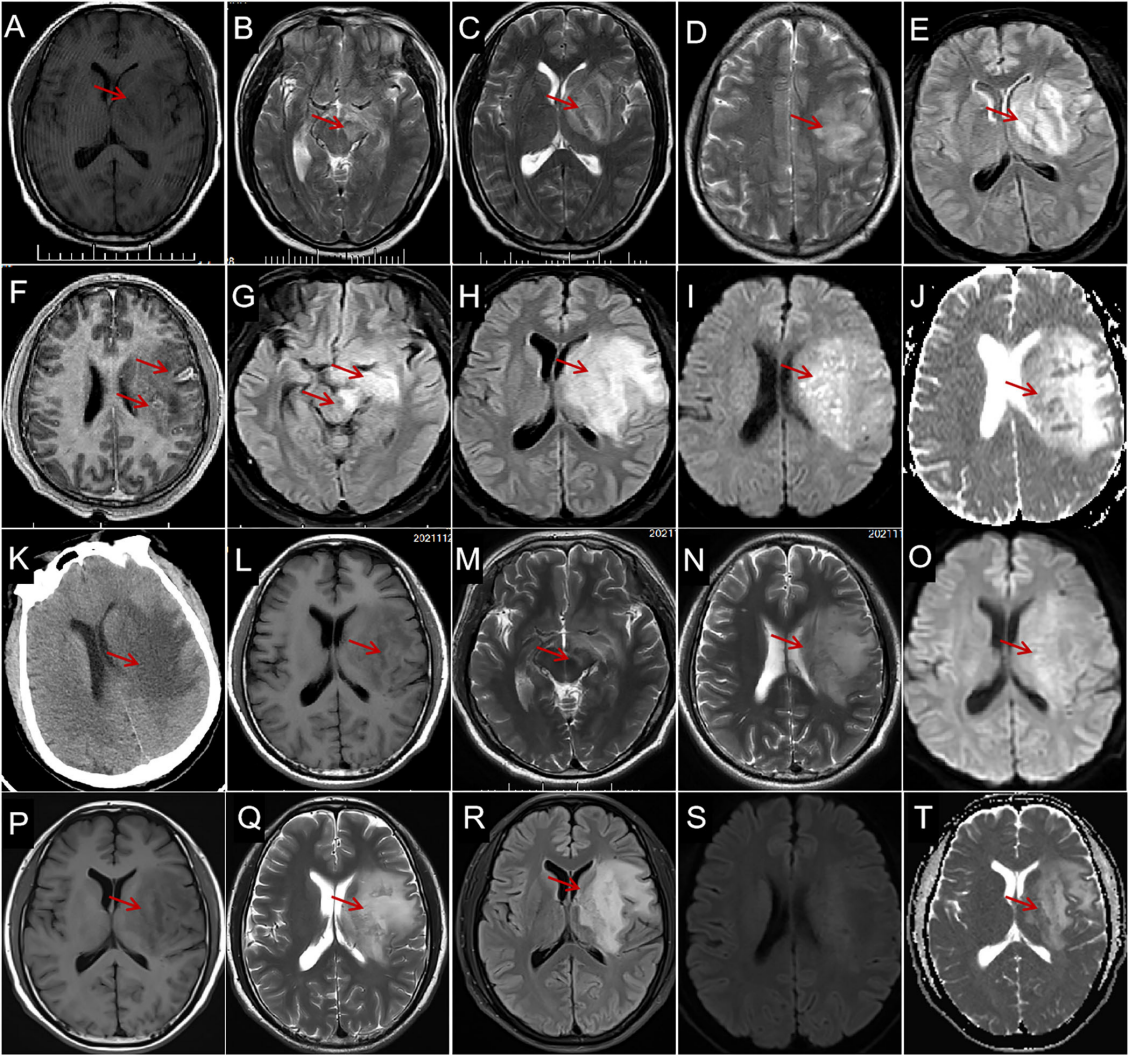
Characteristics and evolution of lesions on brain magnetic resonance imaging (MRI) and computed tomography (CT). **(A)** Brain MRI on day 1 shows a large hypointensity (arrows) in the left basal ganglia and the temporal lobe on axial T1-weighted imaging (WI); a hyperintense region (arrows) in the left midbrain **(B)**, basal ganglia **(C)**, and temporal-parietal lobe **(D)** on T2-WI and fluid-attenuated inversion recovery [FLAIR **(E)**] with space-occupying effect; and individual, small, arc-shaped, ring-shaped lesions are enhanced [**(F)**, arrows]. The MRI on day 5 shows extensive hyperintense lesions enlarged (arrows) in the left midbrain, hippocampus [**(G)**, arrows], basal ganglia, and temporal lobe [**(H)**, arrows] on FLAIR, diffusion-weighted imaging [DWI **(I)**, arrow], and apparent diffusion coefficient [ADC **(J)**, arrow]. CT on day 6 reveals extensive, finger-shaped, low-density areas with space-occupying effects in the left basal ganglia and semioval center [**(K)**, arrow]. MRI on day 34 shows slight hypointensity on T1-WI [**(L)**, arrow], slight hyperintensity on T2-WI [**(M,N)**, arrows], and DWI [**(O)**, arrow], and the space-occupying effect obviously improved. Magnetic resonance imaging on the 25th day after discharge shows hypointensity on T1-WI [**(P)**, arrow], hyperintensity on T2-WI [**(Q)**, arrow], FLAIR [**(R)**, arrow] ADC [**(S)**, arrow], and without diffusion restriction on DWI **(T)**.

Chest CT revealed small ground-glass-like nodules in the posterior segment of the upper lobe of the right lung. Ultrasonography of the liver, bile ducts, pancreas, spleen, and urinary system revealed only mild fatty liver. Brain MRI on day 1 (Figures 1A–F) showed extensive foci involving the left frontoparietal-temporal lobe, basal ganglia, and midbrain.

The patient was initially suspected to have acute cerebral infarction and was administered clopidogrel, argatroban, rosuvastatin, and symptomatic treatment. The patient's condition deteriorated on day 1, and he presented with fever (temperature: 39.2°C) and light coma, followed by a stress peptic ulcer and gastrointestinal bleeding. Neck rigidity was detected on day 2; IV ceftriaxone (2 g/day × 7 days), IV mannitol, IV albumin, and oral esomeprazole magnesium and prednisone (10 mg/day × 7 days) were administered, followed by IV acyclovir (1.5 g/day × 2 weeks) on day 4. However, the anti-infective treatment provided no obvious effect; a repeat MRI on day 5 (Figures 1G–J) and repeat CT (Figure 1K) showed that the lesions had enlarged. Therefore, IV ampicillin sodium, 12 g/day × 5 days, followed by 13.8 g/day × 30 days, was initiated on day 6.

The disturbance of consciousness, fever, and hemiplegia improved on day 10. Afterward, the patient was aphasic but able to walk and lift the right arm on day 28. The MRI on day 34 (Figures 1L–O) showed that the foci obviously improved. The patient was discharged on day 35 with aphasia but independent ambulation. The MRI (Figures 1P–T) on the 25th day after the discharge shows the lesion remained stable. The patient had no discomfort during the 1-month follow-up after discharge and was satisfied with his treatment and recovery.

## Discussion

We report a rare case of a cryptogenic giant brain abscess caused by *Providencia rettgeri* mimicking stroke with a satisfactory outcome after maximum-dose ampicillin therapy. The atypical symptoms and neuroimaging findings in this patient may be related to autoimmune disease and the use of immunosuppressive agents. Opportunistic infections and differentiation of stroke or brain tumor should be carefully considered in patients such as this. Prompt diagnosis and selection of appropriate antibiotic therapy are key to achieving a favorable outcome.

To our knowledge, only two cases of *Providencia stuartii* meningitis have been reported, and in both cases, CSF culture yielded pathogenic bacteria biochemically identified as *Providencia stuartii*; however, no neuroimaging data were provided (9, 10).

### Pathogenesis

In older patients, those with reduced immunity, or patients with long-term indwelling catheters, *Providencia rettgeri* can infect *via* pathogenic adhesion, colonization, invasion, and then urinary tract (7) and intracranial (9) infections. To invade the host cells, an infectious dose of the bacteria is essential (11). The blood–brain barrier (BBB) of the central nervous system (CNS) is an important physiological barrier. Pathogenic bacteria disrupt tight junction proteins and promote BBB permeability (12), and may thwart the autophagic pathway to destroy BBB cellular defense mechanisms (13). The specific mechanism of entry of *Providencia rettgeri* through the BBB is unclear.

### Clinical features

The majority of patients with brain abscesses have predisposing conditions, with half of the cases originating from severe craniocerebral trauma, neurosurgery, otitis, mastoiditis, or sinusitis (2, 14, 15). Brain abscesses can also originate from lung infections (16) and rare oral infections (17). The exact source of the cryptogenic abscess remains unknown in our patient; however, we speculate that this abscess was most likely caused by occult hematogenous spread from an internal focus. The mean duration from the onset of symptoms to the diagnosis of brain abscess is 8.3 days (2), and definitive diagnosis in patients with cryptogenic brain abscess takes longer. Patients with brain abscesses experienced headaches (69%), fever (53%), and focal neurological deficits (48%) (2). This classic triad is observed in only 20% of patients with typical abscess (2), as in our patient who exhibited a stroke-like onset without fever and headache. This could be due to his limited ability to mount an immune response, thereby obscuring the signs typically associated with infection (4). The presence of autoimmune disease and the use of immunosuppressive agents in our patient caused a weakened response to pathogens, which may be an important reason for the hidden clinical manifestations.

### Neuroimaging

The brain MRI in our patient revealed a giant brain abscess in the left temporal-parietal lobe, basal ganglia, and brainstem, with hyperintense lesions on T2-weighted imaging (WI), diffusion-weighted imaging (DWI), apparent diffusion coefficient (ADC), and hypointensity on T1-weighted imaging (T1WI), with space-occupying brain edema, which required differentiation from a brain tumor (18). The appearance of a brain abscess on routine MRI lacks specificity. Diffusion-weighted imaging (DWI) is the most sensitive imaging sequence for brain abscess (19); however, diffusion limitation on MRI can also be seen in high-grade gliomas and primary CNS lymphomas. Diffusion restriction is an uncommon finding in ring-enhancing neoplasms (20, 21). Multimodal MRI is useful in differentiating brain abscesses and tumors.

The most common sites of brain abscesses are in the frontal (5) and temporal lobes (22). An otogenic abscess occurs almost exclusively in the temporal lobe, and sinus infection-related abscess predominantly occurs in the frontal lobe (4). Pyogenic brainstem abscess is rare (23). Abscesses simultaneously involving the brainstem, basal ganglia, and lobes, as in this case, are even more uncommon. The MRI showed no typical intracranial ring-enhancing lesions in this case, which was related either to the absence of abscess capsule formation in the early stage or to the patient's low immune function (4). Enhancing satellite lesions were seen around the abscess (Figure 1F), and multiple lesions of various sizes with annular enhancement have a certain diagnostic value.

### Laboratory analysis

Blood cultures should be performed early in all patients with suspected abscesses, although the positivity rate is not high (14–50%) (4). The CSF analysis may reveal pleocytosis, elevated protein level, and decreased glucose level; however, these parameters will be normal in a significant proportion (16%) of patients (2). The CSF culture is infrequently positive (24%) (2). Metagenomic next-generation sequencing (mNGS) testing of CSF has shown higher sensitivity and specificity relative to conventional microbiological testing in identifying the causative pathogen (24). Metagenomic next-generation sequencing (mNGS) for identifying pathogens is very important, as shown in this case.

### Treatment

*Providencia rettgeri*, in this case, was resistant to ceftriaxone. Therefore, ampicillin sodium was administered for 5 weeks based on the drug sensitivity assay. The duration of antibiotic treatment is usually not < 4 weeks (25). For patients with multiple abscesses or impaired immunity, the required course of antibiotic treatment is 6–8 weeks or longer (4, 25). Prolongation of antibiotic treatment is generally advised as long as the abscess focus is visible on the brain MRI (1). Improvements in our patient's symptoms were observed earlier than brain MRI changes. Timely pathogen identification and effective antibiotics are very important in treating brain abscesses. *Providencia rettgeri* often displays multiple drug resistance (11); therefore, selecting appropriate antibiotics is crucial.

Simple antibiotic therapy is most suitable for patients in good initial clinical condition (Glasgow Coma Scale score >12) and abscesses ≤ 2.5 cm in diameter (25). However, pharmacotherapy alone is rarely effective for abscesses > 3 cm in diameter (26). In our case, the resolution of the abscess—greatly exceeding 3 cm in diameter—was surprising. Although our patient had giant, space-occupying brain lesions, they were deeply situated, involved important functional areas, and did not form an abscess wall. Hence, surgical resection or puncture drainage was not performed.

Neurosurgical treatment of the brain abscess should depend on the location and size of the abscess, the patient's clinical condition, and the chance of achieving successful decompression (15). Surgery should be considered if the condition worsens or there are no clinical and radiological improvements within 1–2 weeks (25). Other surgical indications include cerebellar herniation or periventricular abscess with a risk of rupture (15).

This report is limited in that the origin of the brain abscesses is not clear. The specific process whereby *Providencia rettgeri* evades immunological defenses in the patient with impaired immunity must be investigated.

## Conclusion

Cryptogenic giant *Providencia rettgeri* brain abscesses are rare. Clinicians should consider this opportunistic infection in patients with impaired immunity. Patients with autoimmune diseases or conditions requiring immunosuppressive agents can present atypical symptoms and neuroimaging results that mimic stroke or giant MRI lesions without typical enhancement that must be differentiated from tumors. Metagenomic next-generation sequencing (mNGS) is important in pathogen identification. Rapid diagnosis and appropriate use of antibiotics are key in obtaining a favorable outcome. This study expands the clinical and imaging spectrum of *Providencia rettgeri* meningitis; however, the underlying pathophysiological mechanism requires further study.

## Data availability statement

The original contributions presented in the study are included in the article/supplementary material, further inquiries can be directed to the corresponding author.

## Ethics statement

This report was approved by the Ethics Review Board of the Shenzhen Third People's Hospital (No: 2022-010). The patients/participants provided their written informed consent to participate in this study.

## Author contributions

YZ and DZ were responsible for data collection and writing the first draft. BL and XL provided constructive discussion and translated the manuscript. LC conceived the study, translated the manuscript, and critically revised the manuscript. All authors have read and approved the manuscript.

## Funding

This work was supported by the Shenzhen Second People's Hospital Clinical Research Fund of Guangdong Province High-level Hospital Construction Project (No. 20223357021) and the Initializing Fund of Academic Leaders of Shenzhen Third People's Hospital.

## Conflict of interest

The authors declare that the research was conducted in the absence of any commercial or financial relationships that could be construed as a potential conflict of interest.

## Publisher's note

All claims expressed in this article are solely those of the authors and do not necessarily represent those of their affiliated organizations, or those of the publisher, the editors and the reviewers. Any product that may be evaluated in this article, or claim that may be made by its manufacturer, is not guaranteed or endorsed by the publisher.
