# Corrigendum: Case report: Cryptogenic giant brain abscess caused by *Providencia rettgeri* mimicking stroke and tumor in a patient with impaired immunity

**DOI:** 10.3389/fneur.2022.1113749

**Published:** 2023-01-09

**Authors:** Yu Zhao, Baorong Lian, Xudong Liu, Qizheng Wang, Daxue Zhang, Qi Sheng, Liming Cao

**Affiliations:** ^1^Department of Neurology, Shenzhen Third People's Hospital, Shenzhen, China; ^2^Department of Neurology, The Second Affiliated Hospital, Southern University of Science and Technology, Shenzhen, China; ^3^Shantou University Medical College, Shantou University, Shantou, China; ^4^Department of Neurology, The First Affiliated Hospital of Shenzhen University, Shenzhen, China; ^5^School of Nursing, Anhui Medical University, Hefei, China

**Keywords:** *Providencia rettgeri*, cryptogenic brain abscess, impaired immunity, metagenomic next-generation sequencing, stroke-like onset, brain tumor, case report

In the published article, the affiliation of “Liming Cao” was incorrect. Instead of affiliation 5, they should have affiliation 4.

The authors apologize for this error and state that this does not change the scientific conclusions of the article in any way. The original article has been updated.

